# Chinese Urban Hui Muslims’ Access to and Evaluation of Cardiovascular Diseases-Related Health Information from Different Sources

**DOI:** 10.3390/ijerph15092021

**Published:** 2018-09-15

**Authors:** Lei Yang, Yuping Mao, Jeroen Jansz

**Affiliations:** 1 Erasmus Research Centre for Media, Communication and Culture, Erasmus School of History, Culture and Communication, Erasmus University Rotterdam, 3062 PA Rotterdam, The Netherlands; jansz@eshcc.eur.nl; 2 Department of Communication Studies, College of Liberal Arts, California State University Long Beach, Long Beach, CA 90840, USA; Yuping.Mao@csulb.edu

**Keywords:** Chinese urban Hui Muslims, health information, cardiovascular diseases, access, credibility, preference, minority’s health, culture, health communication

## Abstract

This research aims to identify the sources that urban Hui Muslims access to get health information related to cardiovascular diseases (CVD) and how they evaluate the information from different sources. This paper focuses on health information related to cardiovascular diseases among Hui Muslims. The data was gathered by means of an online survey administered on mobile devices. To put the answers given by Hui Muslims into perspective and make a comparison between Hui Muslims and the Han people, we also gathered information from Han—the dominant group in China. The results showed that Chinese Hui Muslims mostly used mediated sources, while Han people mainly used interpersonal sources. Both Hui Muslims and Han people trusted and preferred health information about cardiovascular diseases provided by health organizations, doctors, and healthcare providers. The information given by religious leaders was trusted the least, although Hui Muslims were significantly more positive about religious authority than the Han people. The current results are relevant for Chinese health information promoters and can help them diffuse CVD health information more effectively to urban Hui Muslims.

## 1. Introduction

This study is aimed at contributing to the development of health communication in mainland China and enhancing knowledge about health communication targeted at Chinese minorities. Although knowledge on health communication among Chinese citizens is increasing, there is limited existing literature on health communication relating to Chinese minorities. This empirical research is concerned with the minority Hui population in China—also called Chinese Hui Muslims—for three main reasons. Firstly, Chinese Hui Muslims are the third biggest group among minorities in China [1]. The 10 million Chinese Hui Muslims are widely distributed in the country [1]. Secondly, Hui minority group is an Islamic group, so they have a different eating habit compared to Han, the population group that forms the majority of the Chinese population. Thirdly, Chinese Hui Muslims are facing a unique health threat by having the highest prevalence of hypertension in China [2]. 

With rapid changes in industrialization, urbanization, and lifestyle in China, morbidities related to being overweight, obese, hypertension, dyslipidaemia, or diabetes present an accelerated trend among the Chinese population [3]. Cardiovascular diseases (CVDs) are now one of the most important diseases influencing the health of Chinese people [2,3]. Existing empirical research shows that Chinese Hui Muslims are more affected by cardiovascular risk factors (CVRFs) compared to other ethnic groups in China [2], which means that there are health disparities between Chinese Hui Muslims and people from other ethnic groups. Thus, corresponding health communication is required among Chinese Hui Muslims. 

Previous research has shown that in multicultural societies, disadvantaged groups are more likely to use specific media (e.g., the internet) to access health information in order to overcome existing social inequalities that limit their access to the information [4]. Chinese Hui Muslims face obstacles to receive effective health information as a result of their different customs and living habits [5]. These obstacles could possibly be addressed by improving health communication. This paper aims to better understand health communication of Chinese urban Hui Muslims by conducting an explorative study on how Chinese urban Hui Muslims access and evaluate CVD-related health information from different sources. McGuire’s communication–persuasion model is applied to understand the online survey data collected from Chinese urban Hui Muslims.

McGuire’s communication–persuasion model [6] is a widely applied theory that presents five key factors that influence communication effectiveness [7]: source, message, channel, receiver, and destination. Among the five fundamental factors of communication development [7], this study mainly focuses on the receiver (Chinese urban Hui Muslims), the message (health information with respect to cardiovascular diseases), and the source (access and evaluation of different sources) to investigate health communication issues among Chinese urban Hui Muslims. 

### 1.1. Receiver: Chinese Hui Muslims in China

In China, 56 ethnic groups have been identified by the central government, and these comprise 55 ethnic minority groups and the Han majority [8]. There are 10 Muslim minority groups among these 55, with the Hui being one of them [1]. The Hui Muslim group is commonly characterized in China as “big distribution, small concentration” [9] as they are dispersed across the so-called Hui autonomous and Hui nonautonomous areas. The latter are also described as scattered and mixed. In the scattered and ethnically mixed areas (e.g., Shenyang City), where Hui Muslims live next to the majority of the population, Hui Muslims are considered almost the same as the majority. While some Hui Muslims have continued to maintain their culture, religious beliefs, and living habits, others have already started to ignore their ethnic traditional culture and do not feel disappointed about the loss of their culture [10]. Compared to the typical Hui minority autonomous area in Northwest China, the Hui Muslim area in the eastern part of China has received limited attention from the media. Therefore, this study concentrates on Hui Muslims living in the eastern part of China, more specifically, in the city of Shenyang. This study will include not only Hui Muslim patients suffering from CVDs and CVRFs but also healthy Hui Muslims. 

Hui Muslims are similar to the majority of the Chinese population in many respects, including customs, language, and culture [1,11]. However, many Hui Muslims follow Islamic dietary laws and take part in religious activities [1]. Even though the Hui culture contains elements from the dominant Han culture, the former is heavily embedded in the Islamic culture [12]. In addition, the social position of Hui Muslims is often vulnerable, for example, their education level is lower than that of the majority of the population [1] and, their income growth is slower than that of the majority group [13]. Previous research has shown that Hui Muslims have a higher prevalence of CVRFs compared to the Han majority group [2]. This research aims to explore health communication differences between the Hui Muslim minority and the Han majority regarding CVD-related information. 

### 1.2. Message: Health Information with Respect to Cardiovascular Diseases

CVDs have been regarded as the leading cause of death worldwide [14] and result in a huge economic burden to individuals and their families [2,15,16]. CVDs are also the leading cause of death in the Chinese population [2,3,15,16]. Based on the Report on Cardiovascular Diseases in China (2014), 290 million Chinese people suffer from CVDs [17], and the prevalence of CVRFs is leading to a further increase in the incidence of cardiovascular diseases [17]. Hypertension, diabetes, dyslipidaemia, overweight/obesity, smoking, and physical inactivity are considered to be the major risk factors for developing CVDs [2,18,19]. Among different ethnic groups, there are distinctions in CVRFs, with a higher incidence among Hui Muslims compared to other ethnic groups in China [2]. Several nationally representative population studies have reported that the prevalence and clustering of major CVRFs have increased in China in the past decades [2,3,15,17]. However, Chinese people do not have enough awareness of how to treat and control CVRFs. For instance, the results of a survey study showed that Chinese people did not have enough awareness of how to control and treat hypertension [20]. Thus, it is imperative for health promoters to convey health information related to CVDs and CVRFs to the Chinese public.

### 1.3. Source: Mediated Sources and Interpersonal Sources

Health communication happens everywhere, not just in medical institutions but also at home and in nonprofit organizations [21]. Chinese people can get health information from professionals [22], hospitals [22], interpersonal networks [23,24], and traditional and new media [22,25,26,27]. 

Chinese people can also access health information from mass media. Mass media channels are all those means of transferring messages that involve a mass medium, such as radio, television, newspapers, and so on, and a few individuals can reach a large audience through one of those mass media channels [28]. Mass media channels are often the most rapid and efficient way to inform audiences about the existence of an innovation [28,29] and have been used as important tools to raise public awareness of health issues [30]. 

For many people, the internet has become an important source for health information and advice [25,31]. A number of people will search health information online before turning to their physicians [31,32]. Social media has improved the connectivity between different individuals and enabled them to have direct online participation. This has direct implications for health communication programs and can also help identify new opportunities whereby social media can be used to influence health of individuals [33,34,35]. In China, many people can access health information from WeChat [36]. Increased access to the internet, combined with strategic uses of social media, can bring public health information to many more people, more quickly and directly than ever before [37]. This is a very relevant development for Hui Muslims in China because they are known to face obstacles in acquiring health information [5].

Individuals often gain health information from their interpersonal networks [26]. Social networks influence many of the lifestyle choices people make in their lives and can also be an essential way of providing support to people [22]. The most important of these relationships are the connection or interaction between an individual and their healthcare providers and social support networks, such as family members and friends [23]. Hui Muslims have specific interpersonal networks that include family members, friends, and imams in mosques. Many Hui Muslims gather at mosques every Friday to do prayers, so this provides a good opportunity for them to communicate with each other about health information. 

A previous research [38] has found that that individuals with younger age, less disability, and higher annual income tend to use mass media rather than interpersonal sources for information. Different sources have different features, so the access frequency can also vary depending on different factors (e.g., gender, age, income, etc.) among different individuals. The following questions are generated to identify the key issues relating to Hui Muslims’ access to different sources:

Research Question 1:

- What sources do Hui Muslims access in Shenyang City to get health information related to CVDs?
- What are the similarities and differences between Hui and Han and between Hui patients and nonpatients in obtaining CVD health information from different sources?
- What factors affect Hui Muslims’ access to these sources (e.g., gender, age, income, etc.)? What are the relationships between these factors and the access?

Individuals have different preferences for information from different sources. Previous research has shown that healthcare providers and the internet are the two sources individuals prefer to obtain health information about a specific disease [32]. A trustworthy source can positively influence an individual’s decision to make healthy choices and may even change their unhealthy behaviors [23]. Previous studies [32,39] have shown that people expressed a higher level of trust for information from their physicians compared to other sources. Thus, in this study, we wanted to know how Hui Muslims evaluate the preference and credibility of CVD health information from different sources. The following questions have been formulated to address this query:

Research Question 2:

- Which sources do Hui Muslims prefer to go to for CVD-related information?
- How do Hui Muslims evaluate the credibility of CVD-related information from different sources?
- What are the similarities and differences between the Hui and Han in preference and credibility of CVD-related information from these sources?

## 2. Materials and Methods 

### 2.1. Pretest and Prestudy

The authors designed the questionnaire in English and then translated it into Mandarin. The questionnaire was pretested by administering it to 10 Chinese bachelor’s and master’s students at a Dutch university. Comments were invited about the clarity of the questions and the ordering. The participants’ feedback resulted in minor changes in the wording and layout of the questionnaire. Subsequently, a larger prestudy was conducted in the city of Urumqi, China with the aim of ascertaining once more whether the questionnaire was clear and whether it was accessible on mobile devices in China. This field test was necessary because mobile devices are the most common way to access the internet in China. The prestudy questionnaire was administered online using Qualtrics. It resulted in 105 completed questionnaires. The answers were analyzed statistically, and the comments were used to improve the instrument’s reliability and validity. The results of the prestudy showed that only minor changes were required.

### 2.2. Sample and Procedures

This study employed a cross-sectional online survey that was administered through Qualtrics and was open from 19 December 2016 to 9 February 2017. It took respondents approximately 15 min to complete the whole questionnaire. The questionnaire was in Mandarin and optimized for mobile devices as that is the most common way to access the internet in China. Snowball sampling was used to find respondents for this study. The first strategy unfolded online by posting an invitation to participate with a link to the survey in popular Chinese social media channels, such as WeChat—an app that is similar to WhatsApp but includes some Facebook functions as well. In addition, the authors used the Chinese QQ groups, which are like WeChat but less popular in China nowadays. This strategy amounted to handing out a paper invitation with a link to the survey among visitors to mosques and parents in a Hui primary school. A total of 738 respondents participated in the survey, and the responses from all of them were included. Incomplete questionnaires were checked. It was established that respondents who did not complete all questions did not differ systematically from the ones that completed all questions; as a result, all responses were kept.

### 2.3. Measurement

#### 2.3.1. Access Frequency

Respondents were asked to report their frequency of access to 8 different sources to get CVD-related health information: (1) internet; (2) television; (3) radio; (4) newspapers and magazines; (5) books, brochures, pamphlets, etc.; (6) family, friends/co-worker (excluding those who work in health-related departments as they belong to source 7); (7) health organizations, doctors, and healthcare providers; and (8) religious organizations and leaders. Respondents were asked to rate the frequency of access on a 5-point Likert scale ranging from “Never” to “Always”.

#### 2.3.2. Evaluation Criteria

Preference of CVD health information from different sources was measured by one single item on a 4-point scale (1 = Not at all; 2 = A little; 3 = Some; 4 = A lot). Trust in health information sources was assessed using a single question—“In general, how much would you trust information related to CVDs and CVRFs from each of the following sources?”—with the eight sources mentioned above included. Respondents were asked to rate their level of trust for each source on a 4-point Likert scale ranging from “as not at all, a little, some, a lot.” A similar single item on trust of health information from different sources was used successfully in the HINTS study, which was conducted in 2014 in the USA (HINTS, 2014; see also Hesse et al., 2005) [32]. 

#### 2.3.3. Demographic and Other Background Variables

The demographic variables included gender, age, ethnic groups, education level, income ranges, insurance status, a geographical variable, and an Islamic eating habit. Apart from demographic variables, there were items about health beliefs and behaviors of respondents—frequency of eating beef, mutton, or inners per week; times of moderate exercise weekly; and frequency of smoking. 

## 3. Results

The dataset was analyzed using statistics as offered in the program SPSS 23 (SPSS Inc., Chicago, IL, USA). The research questions in this paper implied that we focused on both Hui and Han respondents, which enabled us to make comparisons between them.

### 3.1. Descriptive Statistics

The first analysis aimed to show the demographic information and health beliefs and behaviors of survey respondents (see Table 1). Of the Hui Muslim individuals that participated in the survey, 82 (42.9%) were male and 109 (57.1%) were female. The mean age was 40.7 years (Standard Deviation [SD] = 14.0). Of the Hui Muslim respondents, 35.6% had a high school or lower education level and 41.6% Hui Muslim respondents had income of 2001–4000 RMB each month. A large proportion—88.9%—of Hui Muslim respondents had health insurance. Some 39.7% Hui Muslim respondents considered themselves as following Islamic eating habit to some extent, i.e., they preferred Halal food and sometimes drank alcohol.

In terms of the health beliefs and behaviors of respondents, results of *t*-tests showed that Hui respondents (Mean [M] = 2.36) ate more beef, mutton, or inners per week than Han respondents (M = 1.39), *t* (300.7) = −11.0, *p* < 0.01. Han respondents (M = 2.73) smoked more than Hui respondents (M = 2.51), *t* (352.0) = 3.0, *p* < 0.01. Hui (M = 1.58) spent more time on physical activity or exercise than Han (M = 1.41), *t* (383.5) = −2.2, *p* < 0.05. 

### 3.2. Access

The second analysis concerned access and was aimed at finding out the access frequency of different sources of CVD-related health information and making comparisons between Hui and Han respondents and Hui patients and Hui nonpatients. In addition, we wanted to check the factors that affect the access of Hui respondents to these sources. 

Table 2 shows the sources Hui and Han respondents accessed to gather CVD health information. The analysis showed similarities in the sources that Hui and Han accessed. Television was important in both groups— Hui respondents (M = 2.85, SD = 1.09) did access television more often than Han (M = 2.57, SD = 1.01), *t* (411) = −2.71, *p* < 0.01 but the pattern was very similar. Hui participants tended to get CVD health information from television the most (M = 2.85, SD = 1.09), while Han participants tended to get CVD health information from family, friends/co-workers the most (M = 2.64, SD = 1.02). This means Hui participants accessed mediated sources the most, while Han participants accessed interpersonal sources the most. The least used source for both Hui and Han was religious organizations and leaders. Given the embedding of the Islamic faith, it came as no surprise that Hui (M = 1.68, SD = 0.94) scored significantly higher than Han (M = 1.39, SD = 0.73), *t* (312.5) = −3.37, *p* < 0.001 in this measure. 

Hui respondents (M = 1.39, SD = 0.49) looked for CVD health information less than Han respondents (M = 1.50, SD = 0.50), *t* (408.5) = 2.33, *p* < 0.05. Among all the respondents, there were 82 CVD/CVRFs patients (34%) among Han respondents, and there were 83 CVD/CVRFs patients (43.5%) among Hui respondents. Therefore, Hui respondents had a higher percentage of CVD/CVRFs patients than Han respondents, which corresponds with previous research findings [2]. Of the Hui respondents, 137 (72.1%) had family member who had CVDs/CVRFs. Hui CVD/CVRFs patients tended to access internet, health organizations and religious organizations more often than Hui nonpatients (see Table 3). 

A factor analysis assessing frequency of access to CVD health information from the eight sources using a varimax rotation demonstrated a one-factor model with 50.19% of variance explained. Responses on the eight items were found to be highly consistent, as demonstrated by Cronbach’s alpha of =0.86. 

A multiple linear regression was run to test the relation between frequency of access and four demographic factors. Table 4 indicates that the four predictors explained 10.9% of the variance (R Square = 0.109, F (4,158) = 4.82, *p* < 0.001). It was found that gender was significantly positively correlated to the frequency of access to all the sources (β = 0.18, *p* < 0.05), as was age (β = 0.25, *p* < 0.01). Education and income factors could not predict frequency of access to the sources used by Hui participants to gain CVD-related health information. Thus, we can conclude that female and older age Hui participants tended to access CVD-related health information from all the sources more frequently. 

### 3.3. Evaluation of Health Information

The third analysis concerned evaluation and was aimed at identifying how Hui and Han respondents evaluate the credibility of CVD-related health information from different sources. We also wanted to find which sources Hui Muslims individuals prefer to obtain CVD health information and make a comparison between Hui and Han respondents.

Table 5 shows source preference for Hui and Han respondents regarding CVD health information. The results indicated a very similar pattern. Both Hui (M = 2.98, SD = 0.82) and Han (M = 2.92, SD = 0.88) respondents preferred health organizations, doctors, and healthcare providers over other sources to obtain CVD health information. Religious organizations and leaders were the least preferred source of CVD health information for both Hui (M = 2.04, SD = 0.96) and Han (M = 1.70, SD = 0.82) respondents. Even so, Hui respondents (M = 2.04, SD = 0.96) preferred to get CVD health information from religious organizations and leaders (*t* (410) = −3.97, *p* < 0.001) better than Han respondents (M = 1.70, SD = 0.82). 

Table 6 shows how Hui and Han respondents evaluated the credibility of different sources of CVD-related health information. The analysis demonstrated an obvious similarity between Hui and Han. Both Hui (M = 2.86, SD = 0.89) and Han (M = 2.94, SD = 0.92) respondents considered health organizations, doctors, and healthcare providers as the most credible source of obtaining CVD health information. Similarly, both Hui (M = 1.99, SD = 0.87) and Han (M = 1.78, SD = 0.83) respondents considered CVD health information from religious organizations and leaders as the least credible. However, Hui respondents (M = 1.99, SD = 0.87) considered religious organizations and leaders more credible compared to Han respondents (M = 1.78, SD = 0.83), *t* (409) = −2.47, *p* < 0.05. This is because of the Islamic beliefs of the Hui people, which led them to trust religious organizations and leaders better than Han.

## 4. Discussion

This is one of the first studies focusing on health communication issues among Hui Muslims in China. The study employed a survey of Chinese Hui Muslims to examine how they access and evaluate CVD health information from different sources. Ouyang and Pinstrup-Andersen had found that earlier research on health communication related to China mostly focused on the Han people, the majority group in China that accounts for around 91.6% of the country’s population [40]. They also concluded that few papers that have been published in English focused on the remaining 8.4%, which represents 112 million individuals [41] belonging to 55 minority groups [40]. Thus, this study filled a lacuna in research about health communication among Chinese minority groups.

The aim of this study was to identify Hui Muslims’ access to and evaluation of different mediated and interpersonal sources and to see if there were differences between Hui and Han respondents. The Chinese Hui are an Islamic group that has a different culture compared to Han majority in China. We assumed that there would be differences in access to and evaluation of CVD health information from different sources. The results showed one major difference: Hui Muslims accessed mediated sources the most, while Han people accessed interpersonal sources the most. Another notable difference related to the frequency with which religious leaders and organizations were accessed. Hui respondents did this significantly more than Han respondents, which is understandable due to the Islamic beliefs of the Hui minority. Past studies [38] have found that age and income are associated with the use of different sources. However, in this study, we found that gender and age could predict the frequency of access to all sources. Moreover, it did not matter whether Hui Muslims were patients or nonpatients, with gender and age as more important factors than being a patient or not.

In terms of evaluation of CVD health information from various sources, previous research [32,38] has shown that the most trustworthy source of information is physicians. In our study, we got the same result, with Hui respondents considering health organizations, doctors, and healthcare providers the most credible sources to obtain CVD health information. In previous research, a clear preference had been established for using healthcare providers and the internet first when seeking information about a specific disease [32]. In our study, interpersonal networks played the most important role among Hui respondents, with the group preferring health organizations, doctors, and healthcare providers to obtain CVD health information. 

One limitation of this explorative survey is the employment of snowball sampling. This survey was distributed by one of the authors in the city of Shenyang in China, and the author chose snowball sampling, which may have led to respondents from similar backgrounds being recruited. However, as the sample size was substantial and had a decent distribution of age, gender, education, and income levels, we are confident that the results are reliable. A second limitation is that the sample might not be representative of Hui Muslims population in China because it reflects the situation of Hui Muslims in the urban area, and not in the rural area. Future research should focus on a rural area, which would allow a comparison between health communication among Hui Muslims in urban and rural areas.

## 5. Conclusions

Our study highlighted the main sources of CVD health information accessed by Hui Muslims in Shenyang City of China and also studied their evaluation of CVD health information from different mediated and interpersonal sources. The results demonstrate the value of this survey study, which was embedded in McGuire’s communication–persuasion model. Television was the source that Hui Muslims accessed most frequently for CVD health information, so television is an important source for health promoters to diffuse CVD health information among Hui Muslims. The current results are relevant for Chinese health information promoters and may help them diffuse CVD health information more effectively to urban Hui. In addition, the study provides information for future research into health communication among other minority groups in China as our research shows that surveys are an effective tool to obtain data for this kind of study. 

## Figures and Tables

**Table 1:** Descriptive statistics for demographic information and health beliefs and behaviors.

Variables	All N (%)	Hui N (%)	Han N (%)
Ethnic group	458 (100%)	191 (41.7)	241 (52.6)
Mean age, mean (SD)	37.0 (12.8)	40.7 (14.0)	35.0 (11.2)
Gender			
Male	154 (33.7)	82 (42.9)	68 (28.2)
Female	303 (66.3)	109 (57.1)	173 (71.8)
Education			
High school or lower	115 (25.2)	68 (35.6)	41 (17.0)
College degree	103 (22.5)	54 (28.3)	47 (19.5)
Bachelor’s degree	162 (35.4)	58 (30.4)	91 (37.8)
Master’s degree	64 (14.0)	11 (5.8)	51 (21.2)
PhD degree and above	13 (2.8)	0 (0)	11 (4.6)
Income Ranges			
0–2000 RMB	117 (25.8)	60 (31.6)	49 (20.6)
2001–4000 RMB	175 (38.5)	79 (41.6)	86 (36.1)
4001–6000 RMB	84 (18.5)	26 (13.7)	53 (22.3)
6001–8000 RMB	32 (7.0)	11 (5.8)	21 (8.8)
8001–10,000 RMB	18 (4.0)	4 (2.1)	12 (5.0)
10,001 RMB and above	28 (6.2)	10 (5.3)	17 (7.1)
Health Insurance			
Yes	410 (89.7)	169 (88.9)	221 (91.7)
No	47 (10.3)	21 (11.1)	20 (8.3)
Islamic Eating Habit			
Not at all (eat pork)		35 (18.5)	
A little (no pork, but alcohol accepted)		28 (14.8)	
Some (prefer Halal food, drink alcohol sometimes)		75 (39.7)	
A lot (only Halal food, no alcohol)		51 (27.0)	
Frequency of Eating Beef, Mutton, or Inners per week			
Less than twice	238 (52.2)	51 (26.8)	166 (69.7)
2–4 times	116 (25.4)	55 (28.9)	56 (23.5)
5–7 times	63 (13.8)	49 (25.8)	12 (5.0)
More than 7 times	39 (8.6)	35 (18.4)	4 (1.7)
Time Spent on Moderate Exercise			
0–5 h	305 (66.4)	114 (59.7)	170 (70.5)
6–10 h	106 (23.1)	52 (27.2)	52 (21.6)
11–15 h	27 (5.9)	16 (8.4)	10 (4.1)
More than 15 h	21 (4.6)	9 (4.7)	9 (3.7)
Smoking Frequency			
Every day	67 (14.6)	40 (21.1)	26 (10.8)
Some days	29 (6.3)	14 (7.4)	14 (5.8)
Not at all	362 (79.0)	136 (71.6)	201 (83.4)

Note: 1. Sample size varied slightly for each variable because of missing data; 2. Some respondents belonged to other ethnic groups, so the total column does not correspond to the sum of Han and Hui respondents.

**Table 2:** Descriptive analysis using *t*-test for frequency of access to cardiovascular disease (CVD) health information from different sources.

Variables	Hui		Han		*t*-Value
	M	SD	M	SD	
Internet	2.80	1.04	2.63	1.13	NS
Television	2.85	1.09	2.57	1.01	−2.71 **
Radio	2.41	1.18	2.19	1.04	−2.02 *
Newspapers and Magazines	2.45	1.02	2.33	1.07	NS
Books, brochures, pamphlets, etc.	2.47	0.95	2.37	0.99	NS
Family, friends/co-worker	2.83	1.06	2.64	1.02	NS
Health organizations, doctors, and healthcare providers	2.47	1.05	2.43	1.05	NS
Religious organizations and leaders	1.68	0.94	1.39	0.73	−3.37 ***

* *p* < 0.05; ** *p* < 0.01; *** *p* < 0.001; M = Mean; SD = Standard Deviation; NS stands for Not Statistically Significant. All variables range from 1 (Never) to 5 (Always).

**Table 3:** Descriptive analysis using *t*-test for frequency of access to CVD health information from different sources.

Variables	Hui Patients		Hui Non-Patients		*t*-Value
	M	SD	M	SD	
Internet	3.01	1.01	2.65	1.04	2.31 *
Television	2.90	1.11	2.81	1.08	NS
Radio	2.54	1.14	2.33	1.19	NS
Newspapers and Magazines	2.59	1.03	2.35	1.00	NS
Books, brochures, pamphlets, etc.	2.57	0.96	2.40	0.95	NS
Family, friends/co-workers	2.89	0.99	2.78	1.11	NS
Health organizations, doctors, and healthcare providers	2.74	1.11	2.29	0.97	2.87 **
Religious organizations and leaders	1.88	1.10	1.54	0.79	2.19 *

* *p* < 0.05; ** *p* < 0.01; M = Mean; SD = Standard Deviation; NS stands for Not Statistically Significant.

**Table 4:** Regression of frequency of access to all the sources.

	B	SE	β
Gender	2.15	0.89	0.18 **
Age	0.10	0.04	0.25 **
Education	−0.37	0.55	−0.06
Income	0.50	0.35	0.11
*R* ^2^		0.11	
*F*		4.82 ***	

** *p* < 0.01; *** *p* < 0.001.

**Table 5:** Descriptive analysis using *t*-test for source preference for CVD health information.

Variables	Hui		Han		*t*-Value
	M	SD	M	SD	
Internet	2.73	0.80	2.66	0.83	NS
Television	2.67	0.80	2.46	0.79	0.007 **
Radio	2.29	0.85	2.20	0.81	NS
Newspapers and Magazines	2.46	0.82	2.38	0.80	NS
Books, brochures, pamphlets, etc.	2.62	0.77	2.61	0.81	NS
Family, friends/co-workers	2.86	0.73	2.73	0.80	NS
Health organizations, doctors, and healthcare providers	2.98	0.82	2.92	0.88	NS
Religious organizations and leaders	2.04	0.96	1.70	0.82	0.00 ***

** *p* < 0.01; *** *p* < 0.001; M = Mean; SD = Standard Deviation; NS stands for Not Statistically Significant. All variables range from 1 (not at all) to 4 (a lot).

**Table 6:** Descriptive analysis using *t*-test for credibility of sources for CVD health information.

Variables	Hui		Han		*t*-Value
	M	SD	M	SD	
Internet	2.52	0.71	2.45	0.69	NS
Television	2.57	0.74	2.56	0.75	NS
Radio	2.37	0.79	2.28	0.73	NS
Newspapers and Magazines	2.41	0.79	2.41	0.80	NS
Books, brochures, pamphlets, etc.	2.60	0.81	2.66	0.84	NS
Family, friends/co-workers	2.75	0.79	2.70	0.84	NS
Health organizations, doctors, and healthcare providers	2.86	0.89	2.94	0.92	NS
Religious organizations and leaders	1.99	0.87	1.78	0.83	0.000 ***

*** *p* < 0.001; M = Mean; SD = Standard Deviation; NS stands for Not Statistically Significant. All variables range from 1 (not at all) to 4 (a lot).
